# eATP/P2X7R Axis: An Orchestrated Pathway Triggering Inflammasome Activation in Muscle Diseases

**DOI:** 10.3390/ijms21175963

**Published:** 2020-08-19

**Authors:** Chiara Panicucci, Lizzia Raffaghello, Santina Bruzzone, Serena Baratto, Elisa Principi, Carlo Minetti, Elisabetta Gazzerro, Claudio Bruno

**Affiliations:** 1 Center of Translational and Experimental Myology, IRCCS Istituto Giannina Gaslini, 16147 Genova, Italy; chiarapanicucci@gaslini.org (C.P.); lizziaraffaghello@gaslini.org (L.R.); sere.baratto@gmail.com (S.B.); elisaprincipi83@hotmail.it (E.P.); 2 Department of Experimental Medicine, University of Genova, 16126 Genova, Italy; santina.bruzzone@unige.it; 3 Pediatric Neurology Unit, IRCCS Istituto Giannina Gaslini, and University of Genova, 16147 Genova, Italy; minettic@unige.it; 4 Unit of Muscle Research, Experimental and Clinical Research Center Charité Universitätsmedizin and Max Delbrück Research Center, 13125 Berlin, Germany

**Keywords:** eATP, P2X7R, inflammasome, muscular dystrophies, Duchenne Muscular Dystrophy, Sarcoglycanopathies

## Abstract

In muscle ATP is primarily known for its function as an energy source and as a mediator of the “excitation-transcription” process, which guarantees muscle plasticity in response to environmental stimuli. When quickly released in massive concentrations in the extracellular space as in presence of muscle membrane damage, ATP acts as a damage-associated molecular pattern molecule (DAMP). In experimental murine models of muscular dystrophies characterized by membrane instability, blockade of eATP/P2X7 receptor (R) purinergic signaling delayed the progression of the dystrophic phenotype dampening the local inflammatory response and inducing Foxp3^+^ T Regulatory lymphocytes. These discoveries highlighted the relevance of ATP as a harbinger of immune-tissue damage in muscular genetic diseases. Given the interactions between the immune system and muscle regeneration, the comprehension of ATP/purinerigic pathway articulated organization in muscle cells has become of extreme interest. This review explores ATP release, metabolism, feedback control and cross-talk with members of muscle inflammasome in the context of muscular dystrophies.

## 1. Introduction

In muscle cells, intracellular adenosine triphosphate (ATP) is the most abundant energy molecule participating in various signaling pathways (i.e., substrate in kinase-mediated signal transduction, synthesis of cyclic (c)AMP, nucleotides and DNA, etc.). However, ATP is also released in the extracellular space, where it acts as a signaling molecule with paracrine/autocrine effects. It can act as a find me signal from apoptotic cells, where it helps phagocytes to find and safely clear apoptotic cells [1] or as a damage-associated molecular patterns (DAMPs) when it is massively dispersed in the extracellular space in several cell stress—and cell death-inducing conditions, such as mechanical stress, cytotoxic agents, hypoxia or plasma membrane damage [2,3,4]. In this scenario, extracellular ATP (eATP) activates P2 receptors, which include P2XRs and P2YRs, respectively ionotropic and metabotropic receptors. Currently, seven P2XRs have been cloned in mammals (P2XR1-7) [5]. Their activation leads to intracellular modification of cations, such as K^+^, Na^+^ and Ca^2+^ [6]. 

Among the P2XRs, P2X7R responds to high concentration of eATP (>100 μM) [5]. P2X7R is composed by the assembly of three sub-units (homo-trimer) [5]. Each subunit is composed of 595 aminoacids and is formed by a bulky extracellular loop, two transmembrane domains, a short intracellular N-terminus (26 aminoacids) and an extended intracellular C-terminus [7]. In addition to be a non-selective cation channel, P2X7R induces the formation of a plasma membrane pore, permeable to large molecules [8,9] and whose formation can be followed by measurements of the uptake of fluorescent dyes up to 900 kDa, such as ethidium bromide and YO-PRO1 [10,11,12]. The P2X7R’s ability to form the membrane large pore is intrinsic to the receptor itself; however, it has been debated whether P2X7R requires pannexins, annexins or connexins in order to create the pore [13,14,15]. Pore formation occurs upon eATP sustained stimulation and induces cell death via a range of several pathways (e.g., aponecrosis, autosis and autophagy) [9,16,17]. Experimental evidence indicates that, upon ATP stimulation, the opening of P2X7R non-selective cation channel occurs within milliseconds, whereas the formation of a large pore takes place within seconds [18,19].

P2X7R activation results in a plethora of additional downstream effects, including cell proliferation and differentiation [20,21,22], release of pro-inflammatory mediators [23] and activation of metabolic pathways such as the PI3K/Akt/GSK-3 which has been associated with oncogene regulation [24,25] and tumor progression towards malignancy [26,27]. Most of these effects are due to the P2X7R-mediated Ca^2+^ influx or K^+^ efflux and are mediated by the C-terminus tail, which is involved in the proper positioning of the receptor in signaling complexes and in the preservation of an efficient channel function [28,29].

P2XR7 was first described in lymphocytes and macrophages [30,31] and later identified in all immune cell types belonging to both the innate and the adaptive immunity where its role has been extensively elucidated [32,33,34]. Nevertheless, P2X7R has also been detected in non-hematopoietic cells, which can cooperate with the immune cells and contribute to the amplification of the inflammatory processes triggered by eATP [34].

The signal triggered by P2X7R is modulated by the activity of ecto-ATPase enzymatic activities, such as ecto-nucleoside triphosphate diphosphohydrolases (ENTPDases) and ecto-5′-nucleotidase (5′-NT) [4]. More specifically, eATP is hydrolyzed to ADP and AMP, mainly by CD39, an ENTDPase. Subsequently, CD73, a 5′-NT, hydrolyzes AMP to adenosine, which, in turn activates receptors of the P1 ADORA receptor family, represented by four subfamilies (A1, A2A, A2B, and A3) [4]. Stimulation of P1 receptors exerts mostly immunosuppressive and anti-inflammatory effects, contributing to dampen the inflammatory response induced by P2X7R activation [35]. Adenosine deaminase (ADA) regulates adenosine levels by deaminating this nucleoside intoinosine.

Hence, P2X7R-mediated signal activity varies according to the tissue availability of eATP, the receptor density on the cell membrane surface, the co-factors expressed in the specific tissue and the efficiency of hydrolyzing enzymes, which switch off the purinergic signal. Moreover, in the same cellular system, the role for P2X7R may profoundly vary according to the proliferative stage, the functional differentiation phase and the occurrence of pathological derangements.

We will focus on the role of the eATP/P2X7R axis in skeletal muscle cells and muscular tissue in physiological and pathological conditions. Furthermore, we will represent the purinergic signal in muscle as a fine tuned signaling, which activates the inflammasome pathway and requires the orchestrated regulation of different players such as the connexin and pannexin channels, the adenosine receptors and the toll-like receptors (TLRs). 

## 2. P2X7R in Skeletal Muscle Cells and Muscular Tissue at Steady State and Under Pathological Conditions

P2X7R activation in muscle cells shows a dichotomic effect: at physiological low eATP concentrations P2X7R tonic activation is crucial for myoblasts proliferation and differentiation. Under pathological conditions, with increased eATP release and pro-inflammatory state, P2X7R signaling activates cell inflammatory pathways and accelerates cell death.

### 2.1. P2X7R Signaling in Muscle Cells

P2X7R was first detected in C2C12 cell line, an immortalized mouse myoblast cell line [36], in which the purinergic signal is developmentally regulated. Mature myotubes present higher ATP release, P2 receptor surface expression and activity, as well as ATP-hydrolyzing enzyme expression when compared to undifferentiated myoblasts [37]. Among P2XRs, P2X7R protein expression is low in quiescent myoblasts, it increases in proliferating myoblasts and is highly represented in myotubes. In myoblasts, P2X7R stimulates proliferation, which is enhanced by Benzoil ATP (BzATP), an agonist of P2X7R, and inhibited by oxidized ATP (oATP), an antagonist of P2X7R. P2X7R blockade, via oATP, prevents myotubes formation, suggesting a role in full myoblast differentiation [38], although the specific molecular mechanisms still need to be clarified. 

P2X7R’s role in muscle regeneration is controversial. Muscle regeneration and growth are regulated by the muscle stem cells, named satellite cells (SCs), which express the Pax7 transcriptional factor and support the muscle homeostasis through their capacity to activate and to start differentiation and self-renewal. The latter processes are driven by the transcriptional factor MyoD, which regulates the transition of SCs from the state of quiescence toward the activation [39,40,41]. In the *mdx*/P2X7R^−/−^ mice, a double knock-out lacking P2X7R and the dystrophin protein (*mdx*, a spontaneous mouse model of Duchenne Muscular Dystrophy), P2XR7 gene inactivation led to enhanced expression of Myogenin compared to the *mdx* mice [42]. However, P2X7R indirect inhibitory effects on inflammation with the consequent reduction of myofiber degeneration/regeneration cycles hinders the dissection of P2X7R direct action on muscle progenitors in in vivo experiments with dystrophic models. Although the role of P2X7R in muscle regeneration has been evaluated in different neuromuscular disorders, this aspect has been not fully characterized in normal muscle after an acute injury.

The ability to dampen the P2X7R signal is also differently modulated during muscle cell development. Ecto-nucleotidase activity is low in myoblasts and progressively increases during differentiation [37]. Skeletal muscle also presents with a tissue specific ENTPDase, the α-sarcoglycan, expressed in fully differentiated myoblasts, which hydrolyzes only ATP and accounts for the 25% of the total ecto-nucleotidase activity in myoblasts [43]. Contrarily to other ENTPDases [44,45], α-sarcoglycan shows low affinity for ATP (in the mM range) [43], making its role crucial in muscular diseases such as muscular dystrophies characterized by an increased availability of eATP.

### 2.2. P2X7R Triggers the Inflammasome Signaling in Dystrophic Muscle Cells

Primary myoblasts isolated from *mdx* mice show a significantly increase of P2X7R both at the transcript and the protein levels compared to wild types (wt) [46]. Indeed, P2X7R expression is up-regulated by the increased concentration of eATP itself and by exposure to a chronic inflammatory milieu [47]. Accordingly, *mdx* myoblasts demonstrate an enhanced P2X7R-dependent calcium influx and a sustained extracellular signal-regulated kinases 1/2 (ERK1/2) phosphorylation [46,48], which is involved in Reactive Oxygen Species (ROS) production via NADPH oxidases (NOXs) phosphorylation [49,50].

Adinolfi et al. reviewed the P2X7R-driven intracellular inflammatory pathways which range from the leucine-rich repeat (LRR)-containing protein 3 (NLRP3)-inflammasome activation, to the ERK/MAPK kinases and phospholipase A2 phosphorylation and NF-kB transcription factor induction [51]. Muscle cells express the inflammasome platform including NLRP3, a member of the Nucleotide-binding domain (NBD) and leucine-rich repeat (LRR)-containing proteins (NLRs) which contains a pyrin domain 3, the adaptor protein apoptosis-associated speck-like protein containing a caspase recruitment domain (ASC), and the cysteine protease pro-caspase-1. The latter cleaves the cytokines pro-IL-1β and pro-IL-18 into their active forms, facilitating the infiltration of immune cells to damaged or infected tissues and inducing the IL-18 mediated interferon-gamma (IFN-γ) production [52,53,54]. An in vitro challenge with P2X7R agonists of primary skeletal muscle cells isolated from dysferlin-deficient and *mdx* mice was used to simulate the pathological condition occurring in muscular dystrophies [55]. Both myoblasts and myotubes from dysferlin-deficient and *mdx* mice showed a higher basal expression of P2XR, and treatment with Lipopolysaccharides/BzATP (LPS/BzATP) increased the levels of ASC, pro-caspase-1 and pro-IL1b in comparison to wt cells [55]. As a consequence of NLRP3 activation, IL-1β inhibits muscle regeneration and conversely its blocking with recombinant IL-1 receptor antagonist (Kineret^®^) leads to a marked improvement of in vitro muscle differentiation and in vivo amelioration of muscle strength [56,57]. Thus, skeletal muscle cells are equipped with a functional inflammasome pathway and actively participate to the inflammatory response upon P2X7R stimulation. A two-signal model has been suggested for NLRP3 inflammasome turn-on. The priming signal (first step) is provided by TLRs, upon DAMPs or pathogen associated molecular patterns (PAMPs) stimulation. Among DAMPs, the molecules which can trigger TLRs activation are the high-mobility group box 1 (HMGB1), the heat shock proteins (HSPs), as well as extracellular matrix degradation products (hyaluronan, heparan sulphate and biglycan) generated as a result of proteolytic enzymes derived from dying cells [58]. Particular interest has been focused on HMGB1, a nuclear protein that acts as a DNA chaperone in the nucleus and as a signal of tissue damage (DAMP) when extracellularly released, thus contributing to inflammation [59].

TLRs have raised particular attention as potential contributors to muscular dystrophies pathogenesis. As such, TLR2 and TLR4 expression has been reported in myoblasts isolated from wt (C57BL/6J) and dysferlin deficient mice (SJL/J) [55] and *mdx* mice have shown increased HMGB1 together with increased TLR4 sensitivity [60,61].

The first step induces an intracellular downstream signaling leading to NF-kB nuclear translocation with the subsequent upregulation of pro-IL-1β and pro-IL-18 transcripts, together with an increase in NLRP3 expression to a functional level [62]. All TLRs but TLR3, require the Myddosome adaptor protein to initiate the downstream intracellular signaling. The Myddosome complex is an oligomeric signaling platform, consisting of the Myeloid differentiation primary response 88 (MyD88) and IL-1 receptor associated kinases 1 and 4 (IRAK1-4) which recruits the TNFR-associated factor (TRAF) 6 to induce NF-kB nuclear translocation [63,64]. 

**Figure 1:**
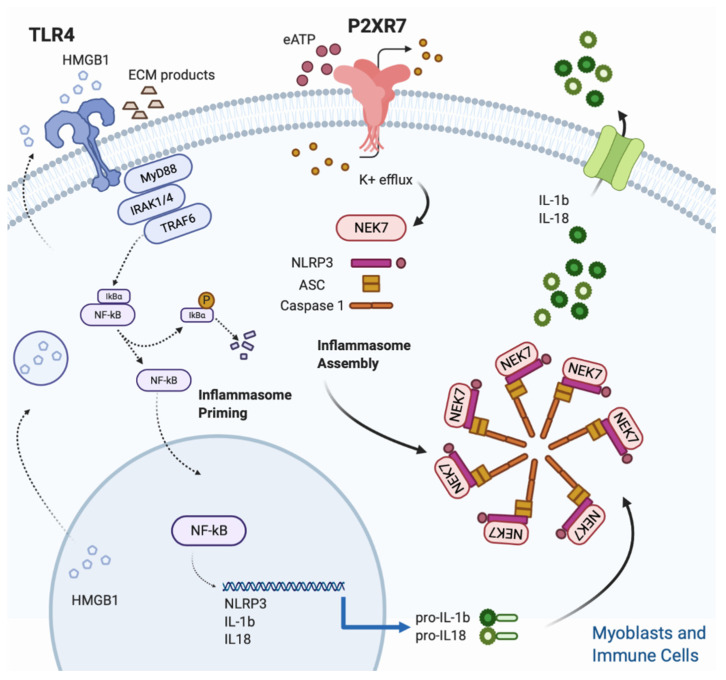
Figure 1. NLRP3 inflammasome activation in skeletal muscle. Skeletal muscle cells are equipped with a functional inflammasome pathway and actively participate to the inflammatory response upon P2X7R stimulation. A two signals model is proposed for NLRP3 inflammasome activation. The Inflammasome priming (first step) is triggered by TLR 4 which in turn is activated upon binding with its cognate ligands such as extracellular matrix degradation (ECM) products and high mobility group Box 1 (HMGB1). HMGB1 is a nuclear protein that acts as a DNA chaperone in the nucleus and as a signal of tissue damage when extracellularly released. Ligand binding and conformational change that occur in the receptor lead to the recruitment of the adaptor protein MyD88, which in turn recruits IRAK4, IRAK1 and IRAK2. IRAK kinases then phosphorylate and activate the protein TRAF6, resulting in IκBα degradation and NF-κB diffusion into the cell nucleus. The nucleus of NFκB activates the transcription of NLRP3, IL-1β and IL-18. The inflammasome assembly (second step) is triggered by the eATP/P2XR7/K^+^ efflux. In particular, intracellular K^+^ efflux, caused by opening of plasma membrane channels (P2X7R included), induces the assembly of the fully active NLRP3 complex (NLRP3-ASC-NEK7-Caspase-1). Finally, caspase-1, cleaves the pro-IL1β and pro IL-18 into their mature forms.

The triggering signal (second step) leads to the NLRP3 inflammasome assembly and is provided by a multitude of stimuli, ranging from K^+^ efflux, ROS generation, mitochondrial and lysosomal damage [62]. Despite the heterogeneity of stimuli listed above, the quick drop of intracellular K^+^ concentration, caused by opening of plasma membrane channels (P2X7R included) and responsible of the assembly of the fully active NLRP3 complex (NLRP3-ASC-NEK7-Caspase-1), is considered the most common event for inflammasome triggering [65,66,67] (Figure 1).

As shown in other cell types [9,16], also myoblasts are susceptible to the detrimental effect of the P2X7R-dependent large pore formation. Indeed, a sustained exposure of *mdx* primary myoblasts to BzATP induces the formation of a large non-selective membrane pore which leads to an increased cell death rate mediated by the heat shock proteins HSP90 and HSPA2 [17].

### 2.3. P2X7R in Dystrophic Skeletal Muscle

**Figure 2:**
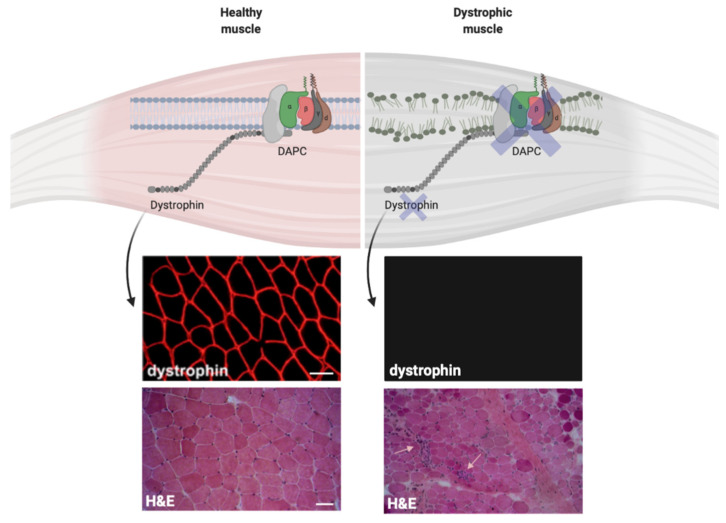
Dystrophin expression influences the plasma membrane stability in muscle cells. Dystrophin expression on the plasma membrane of normal and dystrophic cells. The immunofluorescence stainings for dystrophin show normal dystrophin signal in muscle biopsy collected from healthy control (top left) and the absence of dystrophin in muscle biopsy from Duchenne muscular dystrophy (DMD) patient (top right). The hematoxylin and eosin (H&E) staining show normal muscle architecture in healthy control (bottom left) and tissue architecture disruption in DMD muscle biopsy (bottom right). Small arrows show immune infiltrates within the dystrophic tissue. Scale bars = 50 μm.

Despite the genetic and epidemiologic heterogeneity, several muscular dystrophies share inflammatory features at the muscle biopsy [68,69,70] (Figure 2). The plasma membrane of dystrophic muscular cells shows fragility due to defects on different structural proteins, like dystrophin or sarcoglycans. This induces a chronic release of ATP together with other DAMPs in the extracellular space, thus setting a pro-inflammatory milieu which recruits immune cells within the muscle tissue [71,72]. Specifically, pro-inflammatory M1 macrophages that express the inducible nitric oxide synthase (iNOS) and are specialized in free radicals production, dominate the initial inflammatory response in dystrophic muscle [72]. Although macrophages are the most prevalent inflammatory cell type at early stages, other myeloid cells invade the dystrophic tissue and cooperate with the M1 releasing pro-inflammatory cytokines and chemokines, thus recalling further immune cells, included the adaptive T lymphocytes [72,73]. As such, once triggered, muscle inflammation maintains itself through autocrine/paracrine mechanisms and is involved in the progressive muscle cell death, connective replacement and exhaustion of satellite cell regeneration. In normal muscle, after acute injury, regeneration is promoted by the replacement of M1 with M2 anti-inflammatory macrophages (M2) which express CD163 and support muscle regeneration and repair [72]. Contrarily, in dystrophic muscle, M2 macrophage activation is suppressed by type 1 inflammatory response characterized by increased M1 macrophages, CD4^+^ T helper 1 (Th1), CD8^+^ T cells, natural killer (NK) and CD4^+^ Th17 lymphocytes [74,75,76,77]. In this scenario, given the persistent leakage of DAMPs in the extracellular space, the high expression of P2X7R on the surface of infiltrating immune cells and the active contribution of myoblasts and myotubes to inflammasome activation, the eATP/P2X7R axis represents a possible therapeutic target to ameliorate the dystrophic process. This hypothesis is endorsed by the evidence that P2X7R expression is up-regulated in skeletal muscle tissue isolated from *mdx* mice [46,48] and in muscle biopsies from patients affected by Duchenne muscular dystrophy (DMD) [78].

**Figure 3:**
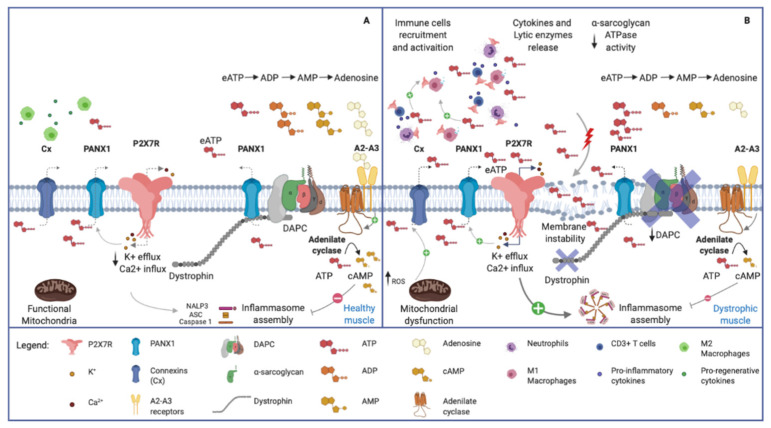
Purinergic signal in normal and dystrophic muscles. (**A**) Healthy skeletal muscle. Note the plasma membrane’s integrity, the normal expression of Dystrophin Associated Protein Complex (DAPC) and the physiological low concentration of eATP. (**B**) Dystrophic skeletal muscle. The membrane instability, due to DAPC alteration, induces chronic release of ATP in the extracellular space, and recalls pro-inflammatory immune cells (i.e., neutrophils, M1 macrophages and CD3^+^ Tcells). eATP, in turn, activates P2X7R receptors on muscular and immune cells, leading to pro-inflammatory citokines release via NLRP3 inflammasome activation. Connexin and Pannexin hemichannels contribute to the release of ATP, being activated respectively by increased ROS production and P2X7R-mediated K^+^ efflux. The high eATP concentration in dystrophic tissue is influenced by the reduced ectoATPase activity mediated by the α-sarcoglycan. Adenosine, acting via A2–A3 receptors, stimulates adenilate cyclase which produces cAMP. The latter compound activates the phosphorylation of NLRP3 reducing its oligomerization and increasing its ubiquitination to be degraded in autophagosomes.

The action of eATP/P2X7R in accelerating muscle cell death in muscular dystrophy is realized through direct and indirect mechanisms. Activation of an abnormal influx of Ca^2+^ leading to myofiber apoptotic events, induction of NLRP3 inflammasome and formation of membrane pores in muscle cells imbricate with an indirect tissue damage from cells of the innate and adaptive immunity infiltrating the tissue (Figure 3). Indeed, P2X7R participates in a feed-forward loop, which contributes to productive T-cell activation [79,80], while P2XR7 blockade determines the functional polarization of naive CD4^+^ cells to adaptive T Regulatory (Tregs) on T-cell receptor stimulation [81,82].

The first in vivo evidence of a beneficial effect of P2X7R inhibition in muscular dystrophies was provided in the mdx model with the broad-spectrum P2X7R antagonist Coomassie Brilliant Blue (CBB) [46]. The treatment determined a significant decrease in the number of revertant fibers in treated mice, indicating a reduced number of degeneration–regeneration cycles. Nevertheless, the hind limb grip strength was significantly reduced in wild-type rats treated with CBB or high doses of a different P2X7 antagonist, compared to vehicle-treated rats, suggesting that in case of future (pre)-clinical trials with P2X7 antagonists, the dose will have to be carefully defined [83].

Similarly, we showed that a short pharmacological in vivo treatment with oATP, a not selective antagonist of P2X7R, improves *mdx* muscular function [78]. The functional beneficial effect exerted by pharmacological P2X7R blockade is associated with a decrease of the pro-inflammatory IL-6 concentration, as well as with a shaping of the T cell composition, with selective increase of the Tregs population. Moreover, Gorecki et al. highlighted a dampening of the *mdx* associated immune response upon in vivo administration of Zidovudine [84], a mainstay of the anti-HIV infection which was shown to exert an anti-purinergic activity [85]. Likewise, P2X7R genetic ablation in *mdx* mice (double knockout *mdx*/P2X7^−/−^ mouse model) showed an overall reduction of muscle inflammatory signature, associated to a decrease of infiltrating leukocytes, and increase in Tregs cell recruitment in muscle [42].

A novel activation of the purinergic pathway was also found in α-sarcoglycan deficient mice (Sgca) [86], the mouse model of α-sarcoglycan-related limb-girdle muscular dystrophy R3 (LGMDR3). Besides being a crucial component of the plasma membrane, α-sarcoglycan retains an ecto-ATPase activity, making the eATP/P2X7R axis activation relevant in the pathogenesis of this muscular dystrophy [43,87]. All together, these results indicate the eATP/P2X7R axis as a promising therapeutic target to dampen the immune response characteristic of muscular dystrophies due to defects in the dystrophin/sarcoglycan complex. The shift of the T-cell pool toward an immunosuppressive phenotype, via purinergic antagonism, is of particular interest since Tregs acutely and chronically infiltrate injured muscle tissue [75,76], exerting a double protective activity. Tregs modulate the myeloid population from a pro- to an anti-inflammatory phenotype and actively promote muscle repair, directly acting on satellite cells, via Areg secretion [75]. 

P2X7R genetic ablation or pharmacological inhibition exerts not only an anti-inflammatory activity but also an anti-fibrotic effect [42] with a decreased concentration of fibrotic mediators such as Transforming Growth Factor (TGF)-β and connective tissue growth factor (CTGF) [78]. Interestingly, P2X7R ablation also ameliorates the cardiac pathology, improves brain performance [42] and bone osteoporotic phenotype [88].

## 3. Other Regulators of P2X7R Signaling

The presence of eATP in the extracellular space depends on a balance between ATP release and ATP-degradation by ecto-enzymes. ATP release occurs through both nonspecific and specific mechanisms. The former as consequence of plasma membrane disruption or cell death, and the latter mediated by different mechanisms, including vesicle and microvesicle exocytosis and transporters/channels, mainly connexin and pannexin hemichannels or the P2X7R itself [4]. In the context of muscular dystrophy, both the mechanisms including the plasma membrane instability, pannexin, connexin and P2X7R work together in controlling the release of ATP. 

Each connexin hemichannel consists of six protein subunits, and each subunit is characterized by four transmembrane domains, connected by two extracellular loops and one intracellular loop, with cytoplasmic carboxyl and amine terminals. Connexins are classified according to the molecular weight (MW) of their subunits, with connexin 43 being the more abundant and involved in many processes. A gap junction is formed by two hemichannels exposed by adjacent cells: through gap juctions, different molecules are exchanged between cells. Instead, a single hemichannel can mediate the release in the extracellular environment of molecules with a MW below 1–2 kDa, including transmitters, as for instance ATP and nicotinamide adenine dinucleotide (NAD). Connexin hemichannels opening can occur at both physiological and pathological conditions and is regulated by different factors, including Ca^2+^ concentration, pH, oxidative stress and mechanical stimulation [4,89,90]. Although connexins and pannexins do not share sequence homology, they possess a similar structure (N and C terminal domain in the cytosol, four transmembrane domain and extra- and intra-cellular loops). The pannexin family in humans is composed of pannexin-1,-2 and -3. Pannexins are only present as hexamerich emichannels, and, as connexin hemichannel, they mediate the extracellular release of small molecules, including nucleotides. Pannexin channels opening is mediated by different conditions, i.e., cleavage of the C-terminal tail by caspases, intracellular Ca^2+^ concentration, plasma membrane depolarization, activation of the P2X7R, redox potential changes, and mechanical stress [4,91]. In the following paragraphs we will review the role of pannexins and connexins in skeletal muscle, and the possibility of targeting these channels in muscle diseases. In addition, we will discuss a novel feedback loop inactivating P2X7R, i.e., the upregulation of MMP2 expression.

### 3.1. Pannexins

Pannexin hemichannels were reported to mediate ATP release upon electrical stimulation [92,93], mechanical stress, and elevated extracellular potassium ion concentration [94]. Pannexin (PANX) 1 and 3 are expressed by myoblasts and SCs and are involved in the regulation of myoblast proliferation, differentiation and skeletal muscle development [92,95,96]. PANX1 levels are dramatically induced during myoblast differentiation, whereas PANX3 is mainly expressed in differentiated human skeletal muscle tissue, and its over-expression inhibits myoblast proliferation and induces their differentiation [95,96]. 

In adult muscle tissue, the PANX1-mediated ATP release after electrical stimulation was shown to potentiate skeletal muscle contraction [97,98] and, as part of a multiprotein complex with the voltage sensor dihydropyridine receptor and Cav1.1, to regulate gene expression, including slow-type Troponin gene [99]. In addition, PANX1 regulates the oxidative state during exercise [100]. PANX3 has also been demonstrated to mediate eATP release [101], likely being involved in contraction and metabolism [96].

In *mdx* mice PANX1 and PANX3 levels are dysregulated, even though in different ways depending on the muscle type [96]. ThePANX3 role in DMD is still to be fully characterized: it has been suggested that PANX3-mediated ATP release may contribute to attraction of monocytes, which may enhance muscle inflammation [94].

Overall, pannexins are important to regulate myoblasts proliferation, differentiation and commitment. Electrical stimulation of myotubes, by causing ATP release, regulates gene expression and muscle plasticity. Furthermore, and importantly to this review, pannexins are critical in enhancing the purinergic signaling to potentiate muscle contraction [102]; in muscular dystrophies, the purinergic signaling seems to be over-activated by the pannexin-mediated eATP release, upon mechanical stress and high concentration of extracellular K^+^. Accordingly, PANX1 and P2X7R were found to functionally and physically interact [103,104,105], thus enabling Pannexins to sense the P2X7R mediated K^+^ efflux and to release ATP with a positive feedback loop primed by P2X7R activation.

### 3.2. Connexins

The role of connexins (Cxs), particularly of Cx43, in different cardiomyopathies, including DMD-associated cardiomyopathies, is well established. Less is known regarding the role of Cx43 and other connexins in skeletal muscle [106]. 

Some members of the connexin family, i.e., Cx39, Cx43 and Cx45, are expressed, along with pannexins, by murine myoblasts and are involved in the regulation of cell proliferation and differentiation [92,107]. In myofibers, connexin expression is down-regulated, and their re-expression occurs during healing processes of injured muscle [107].

Muscle biopsies obtained from patients affected by dysferlinopathy are characterized by expression of Cx40.1 (the Cx hortolog of Cx39 in humans), Cx43 and Cx45. Connexins were expressed by myotubes in a functional form, as demonstrated by uptake assays, together with P2X7R and the Ca^2+^ channel TRPV2. These three channels are likely responsible for a dysregulation in Ca^2+^ homeostasis [108]. Similarly, also myofibers from DMD patients and from *mdx* mice express Cx40.1/Cx39, Cx43 and Cx45 and form functional hemichannels, likely representing key elements in the pathogenesis of the disease [109].

The main isoforms shown to determine ATP release are Cx43, Cx37, Cx26 and Cx36 [110]. Cx43 activation is triggered by changes in the intracellular calcium concentration, cell membrane depolarization, ROS and nitric oxide (NO) [111]. Moreover, in monocytes and macrophages, it has been reported a role of TLR 2-4 in Cx43 activation [111,112]. All these trigger factors have been reported in dystrophic mouse models, making likely an involvement of these channels in ATP release in muscular dystrophies. 

Recently, Nouet and coll. confirmed that Cx43 is over expressed in DMD skeletal muscle, by the use of a mouse *mdx*/wt chimera, a mouse model of DMD manifesting female carriers. Interestingly, in their study, they unveil that Cx43 is not expressed by wt or dystrophinopathic muscle fibers but rather by infiltrating F4/80^+^ mononuclear cells. Cx43 gene inactivation improved histological features of muscular dystrophy in *mdx*/chimera mice suggesting that the development of anti-Cx43 therapies could be beneficial to ameliorate inflammation in DMD [106].

### 3.3. eATP/P2X7R Turning off Mechanisms in Skeletal Muscle

No studies reported the role of the ectoenzymes CD39 and CD73, hydrolyzing extracellular nucleotides, in regulating P2X7R opening in skeletal muscle. Nevertheless, as previously reported, skeletal muscle presents with a tissue specific ENTPDase, the α-sarcoglycan, which carries on the 25% of the total ecto-nucleotidase activity in myoblasts [43].

A novel feedback loop inactivating P2X7R and involving the matrix metalloproteinases (MMPs) has been proposed in muscular cells as in different cell types [113,114]. MMPs are a family of zinc-dependent endopeptidases that are involved in the degradation of various proteins in the extracellular matrix (ECM) [115] and recently, the P2X7R has been unveiled as a MMP2 target [114]. In presence of chronic P2X7R stimulation, MMP-2 activity is up-regulated in skeletal muscle and MMP-2 takes part in an autocrine P2X7R negative feedback signaling. Indeed, high eATP concentration leads to P2X7R large pore formation, which triggers MMP-2 translocation from the cytosol into the extracellular space. The released MMP-2, in turn, exerts its proteolytic function, inactivating the P2X7R [114,116].

### 3.4. Adenosine and ADORA Receptors

Adenosine, the nucleoside derived primarily from the extracellular hydrolysis of adenine nucleotides, is a potent regulator of inflammation and mediates the transition from inflammation to healing, negatively feedbacking eATP effects. Adenosine is active on inflammatory cells (monocytes, macrophages and T cells) by engaging one or more cell-surface receptors. The adenosine receptors belong to the category of purinergic P1-receptors, which have been classified into different subtypes (A1, A2A, A2B, A3) [4]. The cellular effects are mediated by G-proteins. A1 and A3 adenosine receptors exert an inhibitory action on adenylyl cyclase via Gi-proteins, while A2A and A2B receptors stimulate the enzyme by virtue of the Gs-protein. A1 and A2A are high affinity receptors with activity in the low to mid-nanomolar range whereas A2B has a substantially lower affinity and it is triggered by high and rapid eATP accumulation and conversion to adenosine [117]. Adenosine-A2A binding turns-on a wide range of anti-inflammatory pathways among which inhibition of NF-kB signaling and NLRP3 inflammasome, ultimately inducing the switch from M1 to modified M2 macrophage phenotype [118,119]. The role of A2B is not completely clear and the results of current research seem ambiguous. While some studies indicate that Th17 differentiation is stimulated by increasing IL-6 production in dendritic cells upon A2B triggering [120], others sustain that A2B, when stimulated, promotes T cell differentiation in Tregs [121]. Similarly, the A3 receptor function varies from a pro-to an anti-inflammatory and anti-fibrotic effect in different cell types [122,123]. The adenosine A2A and A2B, but not the A1 receptors were found to be present in human skeletal muscle cell membrane and cytosol. The adenosine A1, A2A and A2B receptors were also observed in smooth muscle cells as well as in the endothelial cells [124]. The main intracellular pathway allocated downstream of adenosine A2 receptors in muscular cells is the cAMP/PKA cascade. Stimulation of the adenosine A2 receptors will lead to activation of adenylyl cyclase and the production of cAMP, causing activation of the cAMP-dependent protein kinase (PKA) which in turn may phosphorylate an array of different proteins, e.g., transcription factors such as the cAMP response element binding protein (CREB), thus leading to changes in gene transcription [125].

In cardiac and skeletal muscle adenosine is involved in both the regulation of blood flow [126] and in the synergistic effect of contraction and insulin stimulated glucose uptake [127].

Injured murine skeletal muscle is characterized by increased expression of A2B and A3; however, different cell types including macrophages infiltrating the site of injury express adenosine receptors and a complete study dissecting the involvement of the different cells has not been performed yet [124].

A recent study showed that increased number of systemic CD8/CD26 T cells correlated with muscle strength in DMD patients. The binding of ADA to T cells and the conversion of adenosine to inosine was increased in subject with high CD8/CD26 cells [128], suggesting that prevention of adenosine accumulation may protect muscle from immune damage. However, adenosine/inosine role in muscular dystrophies has not been investigated yet.

## 4. Prospects, Challenges and Future Directions

In muscular dystrophies, the close coupling between stages of inflammation and stages of muscle regeneration is encouraging preclinical approaches that explore whether manipulating inflammation can promote muscle growth and regeneration and can be used in conjunction with gene therapies to ensure efficacy. In this context, eATP and purinergic signaling seem to be ideal harbingers of tissue damage in muscular dystrophies. They activate both innate and adaptive immune responses and inhibit Treg immunosuppressive feedback mechanisms. eATP also exerts a direct toxic effect on muscle cells through P2X receptors. Therefore, the inhibition of purinoreceptors represents a translational potential as a therapeutic strategy for muscular dystrophies. Moreover, due to the fact that the inflammatory response in DMD and sarcoglycanopathies may abrogate the efficacy of gene replacement therapies, therapeutic approaches subduing the immune response, can be used in conjunction with gene therapies to ensure efficacy. However, anti-inflammatory approaches should be considered with extreme caution since inflammation not only causes detrimental effects in muscular tissue but can also support muscle regeneration, as demonstrated in experimental models [129,130]. So far, different P2X7R antagonists have been tested in clinical trials for chronic inflammatory disorders in the past few years (Supplemental Table S1). Although characterized by satisfactory safety and tolerability profile, they were not effective at the designed outcomes. New emerging compounds targeting P2XR7 with high specificity and efficacy were produced [131,132]. Noteworthy, before proposing new P2XR7 antagonists for muscle diseases, the analysis of in vivo P2X7R conditional gene inactivation in muscle cells versus innate/adaptive immune cells can provide relevant and novel inputs. Moreover, targeting other components of the inflammatory machinery such as IL-1β and NLRP3 inflammasome have to be considered. In this regard, IL-1 receptor antagonist has been shown to improve motor functional outcomes in *mdx* mice, although dosage and timing optimization is needed before application in dystrophic patients [57]. In addition it has to be considered that IL-1β production can be mediated by other inflammasomes rather than NLRP3 or by inflammasome-independent pathways; thus inhibitors aimed at IL-1β can result in unintentional immunosuppressive effects. In the past decade, many small molecule inhibitors for NLRP3 inflammasome have been reported and some of them have shown remarkable therapeutic potential, i.e., MCC950, Tranilast and OLT1177 [133]. However, there are no evidences of efficacy of the latter compounds in experimental models of muscular dystrophies. 

As in other biological systems, the eATP/purinergic pathway is finely modulated also in muscle cells, which are adequately fitted with molecules regulating ATP release, metabolism, feedback control and signal activation. Challenging is the development of additional preclinical studies aimed to evaluate the relevance and potential therapeutic effectiveness of other ATP receptors, of the ATP channels, Pannexins and Connexins and of adenosine receptors in dystrophic or inflammatory muscle diseases.

## Supplementary Materials

The following are available online at https://www.mdpi.com/1422-0067/21/17/5963/s1. Supplemental Table S1: anti-P2X7R drugs in clinical trials. List of the available anti-P2X7R drugs which have been proposed in clinical trials.
