# Supplementary material for: eATP/P2X7R Axis: An Orchestrated Pathway Triggering Inflammasome Activation in Muscle Diseases
Source: Int J Mol Sci. 2020 Aug 19;21(17):5963. doi: 10.3390/ijms21175963 (PMC7504480; doi:10.3390/ijms21175963)
Supplement: Supplementary file 1 [file ijms-21-05963-s001.pdf]

Supplemetal Table S1

Target	ID	Title	Disease	Phase	Status
P2XR7	NCT00849134	First Time in Human Study Evaluating the Safety, Tolerability, Pharmacokinetics, Pharmacodynamics and the Effect of Food of Single Assending Doses of GSK1482160.	inflammatory pain	I	Completed
P2XR7	NCT00628095	Study of CE-224,535 A Twice Daily Pill To Control Rheumatoid Arthritis In Patients Who Have Not Totally Improved With Methotrexat	rheumatoid arthritis	II	Completed
P2XR7	NCT00520572	A 6-month Randomised, Double-blind, Open Arm Comparator, Phase IIb, With AZD9056, in Patients With Rheumatoid Arthritis (RA)	rheumatoid arthritis	II	Completed
P2XR7	Eudra-CT Number: 2005- 002319-26 Protocol Number: D8830C00002	Safety and Efficacy of an Oral Inhibitor of the Purinergic Receptor P2X7 in Adult Patients with Moderately to Severely Active Crohn's Disease: A Randomized Placebo-controlled, Double-blind, Phase IIa Study	Crohn disease	II	Completed
P2XR7	NCT02587819	Investigation of the Safety and Tolerability of BSCT (Anti-nf-P2X7) 10% Ointment	basal cell carcinoma	I	Completed
